# Association of L-α Glycerylphosphorylcholine With Subsequent Stroke Risk After 10 Years

**DOI:** 10.1001/jamanetworkopen.2021.36008

**Published:** 2021-11-24

**Authors:** Gyeongsil Lee, Seulggie Choi, Jooyoung Chang, Daein Choi, Joung Sik Son, Kyuwoong Kim, Sung Min Kim, Seogsong Jeong, Sang Min Park

**Affiliations:** 1 Department of Family Medicine, Seoul National University Hospital, Seoul, Korea; 2 Department of Biomedical Sciences, Seoul National University Graduate School, Seoul, Korea; 3 Department of Medicine, Mount Sinai Beth Israel Icahn School of Medicine at Mount Sinai, New York, New York; 4 Department of Family Medicine, Korea University Guro Hospital, Seoul, Korea; 5 National Cancer Control Institute, National Cancer Center, Goyang-si, Gyeonggi-do, Korea

## Abstract

**Importance:**

L-α glycerylphosphorylcholine (α-GPC, choline alphoscerate) is used globally by individuals older than 50 years based on its potential function as a precursor of acetylcholine. However, choline has previously been linked to a higher risk of cardiovascular disease via trimethylamine-*N*-oxide, a metabolite of choline by microbiota.

**Objective:**

To investigate the association between α-GPC use and subsequent 10-year stroke risk.

**Design, Setting, and Participants:**

A population-based, retrospective cohort study was conducted using data from the National Health Insurance Service of South Korea. Participants included men and women aged 50 years or older without underlying stroke or Alzheimer disease (N = 12 008 977).

**Main Outcomes and Measures:**

All participants were divided into whether they were prescribed α-GPC during 2006-2008. α-GPC users were matched with nonusers for all covariates to create a matched cohort. α-GPC use was further divided into durations less than 2, 2 to 6, 6 to 12, and more than 12 months of α-GPC prescriptions. The adjusted hazard ratios (aHRs) and 95% CIs for total stroke, ischemic stroke, and hemorrhagic stroke from January 1, 2009, to January 31, 2018, were calculated by multivariate Cox proportional hazards regression.

**Results:**

A total of 12 008 977 individuals (6 401 965 [53.3%] women) aged 50 years or older were included in the study. The mean (SD) age was 61.6 (9.4) years for nonusers and 68.3 (10.0) years for users, and that of the matching cohort was 68.2 (9.9) years for both groups. Compared with α-GPC nonusers (n = 11 900 100), users (n = 108 877) had a higher risk for total stroke (aHR, 1.46; 95% CI, 1.43-1.48), ischemic stroke (aHR 1.36; 95% CI, 1.33-1.39), and hemorrhagic stroke (aHR, 1.36; 95% CI, 1.28-1.44). After matching for all covariates, α-GPC users had a higher risk for total stroke (aHR, 1.43; 95% CI, 1.41-1.46), ischemic stroke (aHR, 1.34; 95% CI, 1.31-1.37), and hemorrhagic stroke (aHR, 1.37; 95% CI, 1.29-1.46). Increasing intake of α-GPC was associated with a higher risk for total stroke in a dose-response manner.

**Conclusions and Relevance:**

In this cohort study, use of α-GPC was associated with a higher 10-year incident stroke risk in a dose-response manner after adjusting for traditional cerebrovascular risk factors. Future studies are needed to determine the possible mechanisms behind the potential cerebrovascular risk–elevating effects of α-GPC.

## Key Points

**Question:**

Is L-α glycerylphosphorylcholine (α-GPC), a choline analogue, associated with stroke after long-term use?

**Findings:**

In this cohort study of matched cohorts including more than 12 million individuals aged 50 years or older without underlying stroke, Alzheimer disease, or cerebrovascular disease, α-GPC use was significantly associated with a 10-year incident stroke risk in a dose-responsive manner. Individuals using vs not using α-GPC had a 46% higher risk of stroke.

**Meaning:**

The results of this cohort study suggest that the decision to use α-GPC must be carefully weighed with the consideration of potential stroke risk associated with α-GPC.

## Teaser

This cohort study examines the association between use of L-α glycerylphosphorylcholine for 10 years and the incidence of stroke in individuals aged 50 years or older.

## Introduction

The prevalence of dementia among the older population approximately doubles every 5 years,^1^ with 131 million adults worldwide expected to be diagnosed with dementia by 2050.^2^ The goals of dementia management are to treat symptoms associated with cognitive decline and changes in mood and behavior in an attempt to delay progressive cognitive decline.^3^ Acetylcholinesterase inhibitors and *N*-methyl-d-aspartic acid receptor antagonists are used in the treatment of Alzheimer disease.^3^ Other methods of managing dementia could be to enhance brain cholinergic function by supplying choline precursors.^4^ One such precursor, L-α glycerylphosphorylcholine (α-GPC, choline alphoscerate), is now globally used as a prescription or nonprescription drug, based on government certification, which may help manage or prevent dementia progression.

The discrepancies of approving α-GPC as a prescription drug between countries appear to suggest that there may be a lack of sufficient evidence on its efficacy, safety, or both. The only double-blind multicenter trial of α-GPC reported that active treatment using the acetylcholinesterase inhibitors donepezil and α-GPC might slow progressive cognitive decline compared with donepezil treatment alone among 113 participants with Alzheimer disease with cerebrovascular injury after a 12- and 24-month observation period in the Association Between the Cholinesterase Inhibitor Donepezil and the Cholinergic Precursor Choline Alphoscerate in Alzheimer's Disease trial.^5,6^ Apart from this trial, there have been few well-designed studies with large sample sizes to confirm the efficacy of α-GPC.^4^

α-GPC is widely considered to be safe owing to its structure-function feature. Choline, a metabolite of α-GPC,^7^ is an essential nutrient that is naturally present in some foods and supplements,^8^ with potential adverse effects such as fishy body odor, vomiting, excessive sweating and salivation, hypotension, and liver diseases.^8,9^ However, a growing body of evidence suggests that a high plasma choline level is associated with a high risk of cardiovascular disease via trimethylamine-*N*-oxide (TMAO) produced by gut microbiota from choline.^10,11,12,13^ Some studies suggest that TMAO is associated with stroke as well as cardiovascular disease.^14,15,16^

Given that α-GPC is a drug or dietary supplement used for its potential benefits in memory and cognitive function, the possible risk of stroke associated with high levels of choline increases apprehension about its efficacy and safety. We aimed to investigate the association between α-GPC intake and the risk of incident stroke across a 10-year period, including ischemic and hemorrhagic stroke, using the National Health Insurance Service (NHIS) of Korea.

## Methods

### Study Population

The study population was derived from the NHIS. In Korea, all citizens are required to enroll in the NHIS for health insurance, which covers nearly all forms of health services.^17^ The NHIS collects and maintains information on all insured health services for health claim purposes and provides part of the database for research purposes. The health services include information on all inpatient and outpatient department visits, laboratory examinations, diagnostic and surgical procedures, and pharmaceutical prescriptions. Moreover, all citizens aged 40 years or older are eligible for a biannual health-screening examination, which includes the results from a self-reported questionnaire on health behaviors and laboratory blood examinations. The NHIS database has been used in epidemiology studies, and its validity has been described in detail elsewhere.^17,18^

This study was conducted according to the guidelines in the Declaration of Helsinki^19^ and approved by the Seoul National University Hospital Institutional Review Board. All participants were informed of the objective of the survey, and they provided their consent. The NHIS of Korea provided anonymized data according to strict confidentiality guidelines in which the requirement for informed consent was waived.

Among 13 533 281 men and women aged 50 years or older in 2008, we excluded 54 266 individuals who were prescribed α-GPC during 2002-2005. Then, 118 514 individuals with a history of antidementia drug use were also excluded, as well as those who died (n = 196 600) or were diagnosed with stroke (n = 549 558). In addition, 605 366 individuals who had transient ischemic attack (TIA) were excluded, resulting in a final study population of 12 008 977 participants (total cohort). All participants were divided according to α-GPC use within 3 years prior to the index date (2006-2008) and then were followed up from January 1, 2009, until the date of stroke event, death, or January 31, 2018, whichever came earliest. This study followed the Strengthening the Reporting of Observational Studies in Epidemiology (STROBE) guideline.^20^

To minimize the possible confounding effects of covariates, we created a separate cohort after matching each α-GPC user (case) with 10 α-GPC nonusers (controls) according to age, sex, household income, and comorbidities, using the greedy matching method (1:10 age, sex, income, and comorbidity exact matching). Race data were not available. After an α-GPC nonuser was matched with a user, that nonuser was not matched with another user. For sensitivity analysis, 1:1 matching with age, sex, income, and comorbidities was also conducted. Among 108 877 α-GPC new users during 2006-2008 without a history of antidementia drug use, stroke, or transient ischemic attack (TIA), 661 users who were not matched by age, sex, income, and comorbidities with α-GPC nonuser were excluded. Then, 108 216 α-GPC users were 1:10 matched with 1 082 160 α-GPC nonusers also without a history of antidementia drug use, stroke, or TIA, resulting in a matched cohort of 1 190 376 individuals. In addition, for sensitivity analysis, data on 5 425 467 participants who underwent health-screening examinations with additional information on health behaviors and health status were extracted.

### Key Variables

Use of α-GPC was determined during 2006-2008, within 3 years before the index date. Records of pharmaceutical prescription were used to determine α-GPC use, and users were defined as those who were prescribed α-GPC for at least 1 day. Users were further divided into those who had less than 2, 2 to 6, 6 to 12, and more than 12 months of α-GPC intake during 2006-2008. For α-GPC days that extended beyond 2008, only prescription days up to December 31, 2008, were counted.

Stroke was defined as an individual being hospitalized for 2 or more days, with stroke being the main diagnosis according to the *International Statistical Classification of Diseases and Related Health Problems, Tenth Revision (ICD-10)* codes. The *ICD-10* codes for stroke (I60-I69) were in accordance with the guidelines of the American Heart Association.^21^ Along with the risks of stroke, the risks of ischemic stroke (*ICD-10* code I63) and hemorrhagic stroke (*ICD-10* code I61) were also determined. The *ICD-10* codes for ischemic and hemorrhagic stroke were adopted from a previous study that also used the NHIS database to define stroke outcomes.^22^

### Statistical Analysis

Upon comparing the descriptive characteristics of the study population according to α-GPC use, the *t* test was used for continuous variables and χ^2^ test was used for categorical variables. The adjusted hazard ratios (aHRs) and 95% CIs for stroke according to α-GPC use were determined by multivariate Cox proportional hazards regression after adjustments for age (continuous: years), sex (categorical: men and women), household income (categorical: first, second, third, and fourth quartiles), and Charlson Comorbidity Index (CCI) score for the total cohort. The risk for stroke was determined by HRs and 95% CIs without adjustments for the matched cohort because all covariates were matched between α-GPC users and nonusers. Among α-GPC users, the risk of stroke according to α-GPC prescription duration was determined. Competing risk analysis using the cause-specific Cox proportional hazards regression model was conducted for sensitivity analysis. Using death, ischemic stroke (for hemorrhagic stroke assessment), and hemorrhagic stroke (for ischemic stroke assessment) as competing events, the association of α-GPC use with stroke risk and number of days of α-GPC use with stroke were determined. For α-GPC users, aHR values for stroke per 1 IQR increase in α-GPC prescription days were determined. A stratified analysis on the association of α-GPC use with stroke according to subgroups of age, household income, and CCI score was conducted.

In addition, sensitivity analyses on the association of α-GPC use with the risk of stroke among participants who underwent health-screening examinations or after exclusion of participants with stroke events within the first 1 to 4 years of follow-up were conducted. The additional covariates considered upon determination of the association of α-GPC use with the risk of stroke among those who underwent health-screening examinations included smoking (categorical: never, past, and current), alcohol use (categorical: 0, 1-2, 3-4, and ≥5 times per week), physical activity (categorical: 0, 1-2, 3-4, and ≥5 times per week), body mass index (continuous: calculated as weight in kilograms divided by height in meters squared), and presence of hypertension (categorical: yes or no), diabetes (categorical: yes or no), and dyslipidemia (categorical: yes or no). Diabetes was defined as being prescribed antidiabetic medication for diabetes (*ICD-10* codes E11-E14) or having fasting serum glucose levels greater than or equal to 126 mg/dL (to convert to millimoles per liter, multiply by 0.0555). Hypertension was defined as being prescribed antihypertensive medication for hypertension (*ICD-10* code I10) or having blood pressure levels greater than or equal to 140/90 mm Hg. Dyslipidemia was defined as being prescribed statin medication for dyslipidemia (*ICD-10* code E78) or having total cholesterol levels greater than or equal to 240 mg/dL (to convert to millimoles per liter, multiply by 0.0259).

Statistical significance was determined in a 2-sided manner with a threshold of *P* < .05. All data collection and statistical analyses were conducted using SAS Enterprise Guide, version 7.1 (SAS Institute Inc).

## Results

Table 1 depicts the descriptive characteristics of the study population. The number of α-GPC nonusers was 11 900 100 and the number of users was 108 877. The mean (SD) age was 61.6 (9.4) for nonusers and 68.3 (10.0) years for users. In addition to being older, the α-GPC users tended to have lower household income (lowest quartile, 33.7% vs 24.1%), and more comorbidities (CCI score ≥2, 65.7% vs 29.5%) than nonusers in the total cohort (all *P* < .001). Among the matched cohort, there was no significant difference in distribution for the matched variables, including age, sex, income, and CCI score.

**Table 1.** Descriptive Characteristics of the Study Population

Variable	α-GPC use, No. (%)		*P* value^a^
	Nonuser	User	
**Total cohort**			
No. of participants	11 900 100	108 877	NA
Age, mean (SD), y	61.6 (9.4)	68.3 (10.0)	<.001
Sex			
Men	5 568 179 (46.8)	38 833 (35.7)	<.001
Women	6 331 921 (53.2)	70 044 (64.3)	
Household income, quartiles			
1 (highest)	4 124 629 (34.7)	36 273 (33.3)	<.001
2	2 778 519 (23.3)	21 319 (19.6)	
3	2 131 128 (17.9)	14 561 (13.4)	
4 (lowest)	2 865 824 (24.1)	36 724 (33.7)	
Charlson Comorbidity Index score			
0	5 496 687 (46.2)	13 812 (12.7)	<.001
1	2 898 511 (24.4)	23 486 (21.6)	
≥2	3 504 902 (29.5)	71 579 (65.7)	
**Matched cohort**			
No. of participants	1 082 160	108 216	NA
Age, mean (SD), y	68.2 (9.9)	68.2 (9.9)	>.99
Sex			
Men	386 170 (35.7)	38 617 (35.7)	>.99
Women	695 990 (64.3)	69 599 (64.3)	
Household income, quartiles			
1 (highest)	361 900 (33.4)	36 190 (33.4)	>.99
2	212 300 (19.6)	21 230 (19.6)	
3	144 870 (13.4)	14 487 (13.4)	
4 (lowest)	363 090 (33.6)	36 309 (33.6)	
Charlson Comorbidity Index score			
0	138 120 (12.8)	13 812 (12.8)	>.99
1	234 810 (21.7)	23 481 (21.7)	
≥2	709 230 (65.5)	70 923 (65.5)	

Abbreviations: α-GPC, L-α glycerylphosphorylcholine; NA, not applicable.

^a^ *P* values calculated by the *t* test for continuous variables and χ^2^ test for categorical variables.

The results of the association of α-GPC use with the risk of stroke within the total and matched cohorts are reported in Table 2. Compared with α-GPC nonusers, individuals who were prescribed α-GPC had a higher risk for total stroke (aHR, 1.46; 95% CI, 1.43-1.48), ischemic stroke (aHR, 1.36; 95% CI, 1.33-1.39), and hemorrhagic stroke (aHR, 1.36; 95% CI, 1.28-1.44). α-GPC users had a higher risk for total stroke compared with nonusers among men (aHR 1.56; 95% CI, 1.52-1.60) and women (aHR 1.39; 95% CI, 1.36-1.42). Within the matched cohort, α-GPC use was associated with a higher risk for total stroke (aHR, 1.43; 95% CI, 1.41-1.46), ischemic stroke (aHR, 1.34; 95% CI, 1.31-1.37), and hemorrhagic stroke (aHR, 1.37; 95% CI, 1.29-1.46). Similar associations were observed upon competing risk analysis via cause-specific Cox proportional hazards regression model (eTable 1 in the Supplement).

**Table 2.** Adjusted Hazard Ratios for Stroke According to α-GPC Use^a^

Variable	Total		Men		Women	
	Nonuser	User	Nonuser	User	Nonuser	User
**Total cohort**						
Total stroke						
Events	745 589	14 138	362 154	5426	383 435	8712
Person-years	107 830 473	870 174	49 775 056	295 203	58 055 417	574 970
aHR (95% CI)	1 [Reference]	1.46 (1.43-1.48)	1 [Reference]	1.56 (1.52-1.60)	1 [Reference]	1.39 (1.36-1.42)
Ischemic stroke						
Events	446 469	8342	233 444	3311	213 025	5031
Person-years	107 830 473	870 174	49 775 056	295 203	58 055 417	574 970
aHR (95% CI)	1 [Reference]	1.36 (1.33-1.39)	1 [Reference]	1.44 (1.39-1.49)	1 [Reference]	1.30 (1.27-1.34)
Hemorrhagic stroke						
Events	69 376	1089	35 767	413	33 609	676
Person-years	107 830 473	870 174	49 775 056	295 203	58 055 417	574 970
aHR (95% CI)	1 [Reference]	1.36 (1.28-1.44)	1 [Reference]	1.39 (1.26-1.53)	1 [Reference]	1.34 (1.24-1.44)
**Matched cohort**						
Total stroke						
Events	101 067	14 056	36 623	5407	64 444	8649
Person-years	8 939 584	867 451	3 072 198	294 503	5 867 386	572 948
aHR (95% CI)	1 [Reference]	1.43 (1.41-1.46)	1 [Reference]	1.54 (1.50-1.58)	1 [Reference]	1.38 (1.34-1.41)
Ischemic stroke						
Events	63 895	8295	24 185	3299	39 710	4996
Person-years	8 939 584	867 451	3 072 198	294 503	5 867 386	572 948
aHR (95% CI)	1 [Reference]	1.34 (1.31-1.37)	1 [Reference]	1.42 (1.37-1.48)	1 [Reference]	1.29 (1.25-1.33)
Hemorrhagic stroke						
Events	8139	1083	3102	412	5037	671
Person-years	8 939 584	867 451	3 072 198	294 503	5 867 386	572 948
aHR (95% CI)	1 [Reference]	1.37 (1.29-1.46)	1 [Reference]	1.38 (1.25-1.53)	1 [Reference]	1.37 (1.26-1.48)

Abbreviations: α-GPC, L-α glycerylphosphorylcholine; aHR, adjusted hazard ratio.

^a^ Adjusted hazard ratios calculated by Cox proportional hazards regression after adjustments for age, sex, household income, and Charlson Comorbidity Index score.

The Figure depicts the risk of stroke according to α-GPC prescription duration among α-GPC users. Compared with those who were prescribed α-GPC for less than 2 months, individuals with 2 to 6 (aHR, 1.13; 95% CI, 1.09-1.18), 6 to 12 (aHR, 1.18; 95% CI, 1.12-1.24), and more than 12 (aHR, 1.36; 95% CI, 1.29-1.43) months of α-GPC use had a higher risk for stroke in a dose-response manner (*P* < .001). Similarly, participants with 2 to 6 (aHR, 1.10; 95% CI, 1.05-1.17), 6 to 12 (aHR, 1.15; 95% CI, 1.08-1.23), and more than 12 (aHR, 1.37; 95% CI, 1.28-1.46) months of α-GPC use had a higher risk for ischemic stroke than those with less than 2 months of use (*P* < .001 for trend). In addition, compared with users with less than 2 months of use, those with 2 to 6 (aHR, 1.23; 95% CI, 1.06-1.44), 6 to 12 (aHR, 1.07; 95% CI, 0.89-1.29), and more than 12 (aHR, 1.34; 95% CI, 1.11-1.61) months of α-GPC prescription had a higher risk of hemorrhagic stroke (*P* = .002). Similar associations were observed upon competing risk analysis via cause-specific Cox proportional hazards regression model (eFigure in the Supplement).

**Figure.**
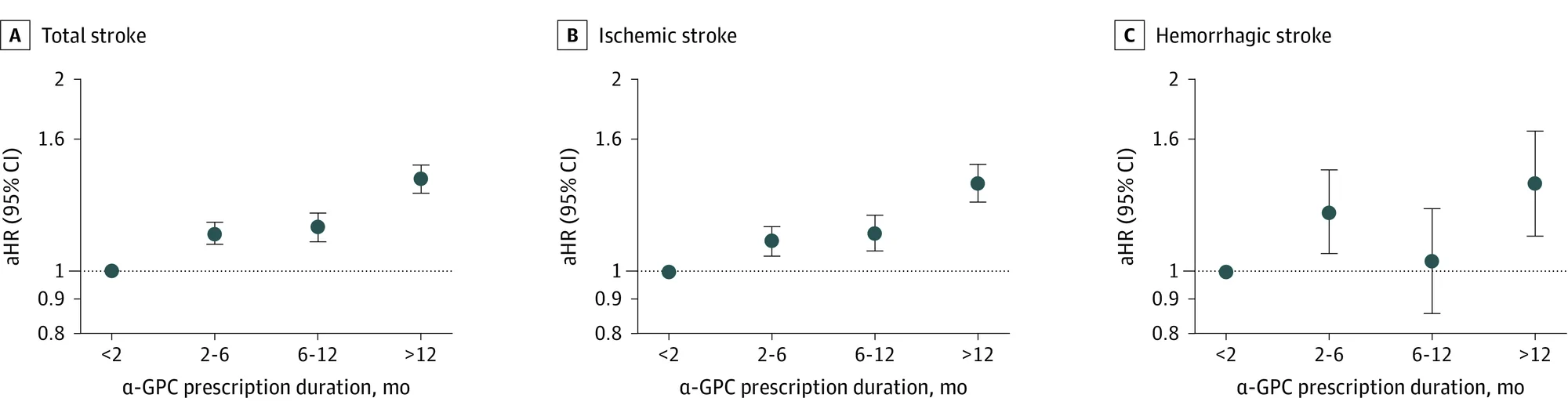
Adjusted Hazard Ratios (aHRs) for Stroke According to L-α Glycerylphosphorylcholine (α-GPC) Duration of Prescriptions A, Total stroke (*P* < .001). B, Ischemic stroke (*P* < .001). C, Hemorrhagic stroke (*P* = .002). The aHRs were calculated by Cox proportional hazards regression after adjustments for age, sex, household income, and Charlson Comorbidity Index score. Error bars indicate 95% CIs; dashed lines, reference values of 1.0.

The results of the stratified analysis on the association of α-GPC use with the risk of total stroke according to subgroups of household income and CCI score within the total cohort are given in Table 3. Users within the upper (aHR, 1.44; 95% CI, 1.41-1.48) or lower (aHR, 1.49; 95% CI, 1.46-1.53) half of the household income levels had a higher risk for total stroke. Users of α-GPC with a CCI score less than or equal to 1 (aHR, 1.52; 95% CI, 1.47-1.56) or greater than or equal to 2 (aHR, 1.49; 95% CI, 1.46-1.52) both had a higher risk for total stroke than nonusers. Similar results were observed for men and women.

**Table 3.** Stratified Analysis on the Association of α-GPC With Total Stroke Risk Among Subgroups^a^

Variable	aHR (95% CI)					
	Total		Men		Women	
	Nonuser	User	Nonuser	User	Nonuser	User
Age, y						
<65	1 [Reference]	1.79 (1.73-1.86)	1 [Reference]	1.85 (1.76-1.95)	1 [Reference]	1.73 (1.65-1.82)
≥65	1 [Reference]	1.52 (1.49-1.55)	1 [Reference]	1.66 (1.61-1.72)	1 [Reference]	1.45 (1.41-1.48)
Household income						
Upper half	1 [Reference]	1.44 (1.41-1.48)	1 [Reference]	1.51 (1.46-1.57)	1 [Reference]	1.39 (1.35-1.44)
Lower half	1 [Reference]	1.49 (1.46-1.53)	1 [Reference]	1.64 (1.58-1.71)	1 [Reference]	1.41 (1.36-1.45)
Charlson Comorbidity Index score						
≤1	1 [Reference]	1.52 (1.47-1.56)	1 [Reference]	1.59 (1.51-1.67)	1 [Reference]	1.47 (1.41-1.54)
≥2	1 [Reference]	1.49 (1.46-1.52)	1 [Reference]	1.62 (1.57-1.67)	1 [Reference]	1.43 (1.39-1.46)

Abbreviations: α-GPC, L-α glycerylphosphorylcholine; aHR, adjusted hazard ratio.

^a^ Adjusted hazard ratios calculated by Cox proportional hazards regression after adjustments for age, sex, household income, and Charlson Comorbidity Index score.

Results of the sensitivity analysis on the association of α-GPC use with the risk of stroke among individuals who underwent health-screening examinations or after excluding participants with stroke events within the first 1 to 4 years of follow-up within the total cohort are presented in Table 4. After additional adjustments for health behaviors and health status, users had a higher risk for total stroke (aHR, 1.48; 95% CI, 1.43-1.52), ischemic stroke (aHR, 1.40; 95% CI, 1.35-1.46), and hemorrhagic stroke (aHR 1.41; 95% CI, 1.27-1.56) than α-GPC nonusers. Users also had a higher risk for total stroke (aHR, 1.34; 95% CI, 1.31-1.37), ischemic stroke (aHR, 1.26; 95% CI, 1.22-1.30), and hemorrhagic stroke (aHR, 1.27; 95% CI, 1.17-1.39) after excluding those with stroke events within the first 4 years of follow-up.

**Table 4.** Sensitivity Analysis on the Association of α-GPC Use With Stroke Risk^a^

Variable	aHR (95% CI)					
	Total		Men		Women	
	Nonuser	User	Nonuser	User	Nonuser	User
**Additional adjustments^b^**						
Total stroke	1 [Reference]	1.48 (1.43-1.52)	1 [Reference]	1.53 (1.47-1.60)	1 [Reference]	1.44 (1.39-1.49)
Ischemic stroke	1 [Reference]	1.40 (1.35-1.46)	1 [Reference]	1.44 (1.37-1.53)	1 [Reference]	1.36 (1.30-1.43)
Hemorrhagic stroke	1 [Reference]	1.41 (1.27-1.56)	1 [Reference]	1.40 (1.19-1.64)	1 [Reference]	1.40 (1.23-1.60)
**Latent period, time of follow-up^c^**						
Total stroke						
1 y	1 [Reference]	1.41 (1.39-1.44)	1 [Reference]	1.50 (1.46-1.55)	1 [Reference]	1.36 (1.33-1.39)
2 y	1 [Reference]	1.37 (1.35-1.40)	1 [Reference]	1.46 (1.41-1.51)	1 [Reference]	1.32 (1.29-1.35)
3 y	1 [Reference]	1.36 (1.33-1.39)	1 [Reference]	1.44 (1.39-1.49)	1 [Reference]	1.31 (1.27-1.34)
4 y	1 [Reference]	1.34 (1.31-1.37)	1 [Reference]	1.40 (1.35-1.46)	1 [Reference]	1.30 (1.26-1.34)
Ischemic stroke						
1 y	1 [Reference]	1.32 (1.29-1.35)	1 [Reference]	1.38 (1.33-1.44)	1 [Reference]	1.26 (1.22-1.30)
2 y	1 [Reference]	1.29 (1.25-1.32)	1 [Reference]	1.35 (1.30-1.41)	1 [Reference]	1.23 (1.19-1.27)
3 y	1 [Reference]	1.28 (1.24-1.31)	1 [Reference]	1.35 (1.29-1.41)	1 [Reference]	1.22 (1.17-1.26)
4 y	1 [Reference]	1.26 (1.22-1.30)	1 [Reference]	1.31 (1.25-1.38)	1 [Reference]	1.21 (1.16-1.26)
Hemorrhagic stroke						
1 y	1 [Reference]	1.32 (1.24-1.41)	1 [Reference]	1.34 (1.20-1.49)	1 [Reference]	1.30 (1.20-1.42)
2 y	1 [Reference]	1.30 (1.21-1.39)	1 [Reference]	1.34 (1.19-1.51)	1 [Reference]	1.27 (1.16-1.39)
3 y	1 [Reference]	1.29 (1.20-1.40)	1 [Reference]	1.32 (1.16-1.50)	1 [Reference]	1.27 (1.15-1.49)
4 y	1 [Reference]	1.27 (1.17-1.39)	1 [Reference]	1.24 (1.07-1.43)	1 [Reference]	1.29 (1.16-1.43)

Abbreviations: α-GPC, L-α glycerylphosphorylcholine; aHR, adjusted hazard ratio.

^a^ Participants included those who underwent health-screening examinations, after excluding participants with stroke events within the first 1 to 4 years of follow-up within the total cohort.

^b^ Adjusted hazard ratios calculated by Cox proportional hazards regression after adjustments for age, sex, household income, Charlson Comorbidity Index, smoking, alcohol use, physical activity, body mass index, diabetes, hypertension, and dyslipidemia. Diabetes was defined as being prescribed antidiabetic medication for diabetes (*International Statistical Classification of Diseases and Related Health Problems, Tenth Revision *[*ICD-10*] codes E11-E14) or having fasting serum glucose levels greater than or equal to 126 mg/dL (to convert to millimoles per liter, multiply by 0.0555). Hypertension was defined as being prescribed antihypertensive medication for hypertension (*ICD-10* code I10) or having blood pressure levels greater than or equal to 140/90 mm Hg. Dyslipidemia was defined as being prescribed statin medication for dyslipidemia (*ICD-10* code E78) or having total cholesterol levels greater than or equal to 240 mg/dL (to convert to millimoles per liter, multiply by 0.0259).

^c^ Adjusted hazard ratios calculated by Cox proportional hazards regression after adjustments for age, sex, household income, and Charlson Comorbidity Index score.

After 1:1 matching, the risk for total stroke, ischemic stroke, and hemorrhagic stroke was significantly higher among α-GPC users compared with α-GPC nonusers (eTable 2 in the Supplement). In addition, eTable 3 in the Supplement depicts the risk for stroke per 1-IQR increase in α-GPC prescription days among those who were prescribed α-GPC. For a 1-IQR increase in α-GPC prescription days, the risk for total stroke (aHR, 1.09; 95% CI, 1.07-1.10), ischemic stroke (aHR, 1.09; 95% CI, 1.07-1.10), and hemorrhagic stroke (aHR, 1.06; 95% CI, 1.02-1.11) were significantly increased.

## Discussion

In this large cohort study of more than 12 million men and women aged 50 years or older who did not have underlying stroke, TIA, or Alzheimer disease at baseline, α-GPC use was associated with a higher risk of stroke within 10 years in a dose-response manner after adjusting for traditional cerebrovascular risk factors. To our knowledge, this is the first study to investigate the long-term adverse events of α-GPC use.

Although, to our knowledge, no trials have examined the effect of α-GPC on stroke, previous studies have determined the association between TMAO from dietary choline or lecithin and stroke.^15,16,23^ This outcome may be relevant as α-GPC is converted to free choline through hydrolysis in the gut mucosa,^7^ and increased plasma levels of choline reflect the absorption of α-GPC.^24^ A nested case-control study reported that higher TMAO levels appeared to be linked to an increased risk of the first stroke among patients with hypertension.^16^ Another case-control study also reported that patients with ischemic stroke had higher levels of TMAO, suggesting that higher TMAO levels were associated with an increased risk of the first stroke.^23^ A multicenter prospective cohort study reported that increased TMAO levels were associated with an increased risk of new ischemic brain lesions among patients with severe carotid artery stenosis.^15^ A prediction model reported that TMAO was an independent predictor of ischemic stroke among patients with atrial fibrillation.^25^ We also noted that α-GPC use appears to be dose-dependently associated with stroke risk.

The mechanism by which TMAO induces stroke is not well understood; however, it is widely established that the TMAO pathway promotes the development of atherosclerosis and thrombosis.^11,26,27^ First, a metabolomics study reported that dietary supplementation of mice with choline, betaine, and TMAO activates upregulation of multiple macrophage scavenger receptors and subsequent accumulation of cholesterol and foam cells.^11^ Second, an atherogenesis study reported that TMAO promotes proinflammatory changes in arterial walls through mitogen-activated protein kinase and nuclear factor-kB signaling.^26^ Third, TMAO could directly contribute to platelet hyperreactivity and enhanced thrombosis.^27^ Taken together, these mechanisms suggest that the α-GPC dose-dependent development of ischemic stroke is plausible. However, these explanations assume that increased TMAO levels are caused by choline but not α-GPC itself, although high plasma choline induced by α-GPC has been confirmed.^24^ Further studies on the biological and pharmacological mechanisms of how α-GPC could increase the risk of stroke are warranted.

In addition, we noted that the risk of hemorrhagic stroke increased with α-GPC use. A previous study reported that a high level of TMAO is associated with the risk of hemorrhagic stroke among patients with hypertension.^16^ The mechanisms behind hemorrhagic stroke include lipohyalinosis of small arteries and fibrinoid necrosis induced by long-standing hypertension, followed by formation of small microaneurysms (Charcot-Bouchard aneurysms) that subsequently rupture.^28^ When endothelial dysfunctionlike fibrinoid necrosis occurs, excessive reactive oxygen production and inflammation are inevitable.^29^ Additive inflammation by TMAO could be the accelerator of hemorrhagic stroke. It is plausible that the previous study showed patients with hypertension were more likely to have a hemorrhagic stroke than an ischemic stroke according to baseline TMAO levels.^16^ We also found that the development of hemorrhagic stroke was associated with the duration of α-GPC use, possibly because some—but not all patients—have hypertension. Nevertheless, although significant, this association needs to be further noted.

### Limitations

This study has limitations. First, α-GPC users were older and had more comorbidities than α-GPC nonusers, which suggests that α-GPC users may already have subclinical atherosclerotic changes. However, the risk of stroke increased with α-GPC use even after not only 1:10 but also 1:1 matching for age, sex, income, and comorbidity. We excluded patients with TIA at baseline in an attempt to remove individuals with potential subclinical atherosclerotic changes, which could have confounded the association of α-GPC with the risk of stroke. Nevertheless, some individuals were still prescribed α-GPC, which might come from broad indications of the insurance coverage of α-GPC in South Korea, including dementia or secondary symptoms of cerebrovascular defects, changes in mood and behaviors, and senile depression. Therefore, α-GPC was frequently prescribed for the purpose of possibly preventing cognitive decline in the older cohort, even without dementia.

Second, the development of stroke confirmed by hospitalization for 2 days or more with the relevant *ICD-10* codes may have led to an underestimation of the actual number of stroke events. Nonetheless, a previous study noted that identifying cardiovascular disease cases using diagnostic codes from a claims database was more than 80% accurate.^30^ In addition, because the target population is older individuals, it was expected that many deaths would occur, in which case death events may act as competing events for the development of stroke. However, the results of a competing risk analysis after treating death as a competing event also showed that the use of α-GPC was consistently associated with an increased risk for stroke. Despite these findings, future studies that use cases of verified stroke events from medical records would be beneficial.

## Conclusions

In this cohort study, use of α-GPC was associated with 10-year subsequent stroke in a dose-response manner after adjustment for traditional cerebrovascular risk factors. To our knowledge, this is the first study to examine long-term adverse events of α-GPC. We suggest that manufacturers producing α-GPC adequately report safety issues, and postmarketing surveillance should be conducted by drug approval authorities.

## Notes

Supplement. **eTable 1.** Hazard Ratios for Stroke According to α-GPC Use With Competing Risk Analysis Via Cause-Specific Hazard Model Regression **eFigure.** Hazard Ratios for Stroke According to α-GPC Prescription Amount Among Those Who Were Prescribed α-GPC With Competing Risk Analysis Via Cause-Specific Hazard Model Regression **eTable 2.** Hazard Ratios for Stroke According to α-GPC Use After 1:1 Exact Matching **eTable 3.** Hazard Ratios for Stroke per 1 Interquartile Range Increase in α-GPC Prescription Days Among Those Who Were Prescribed α-GPC
