# Errors in Methods and Article Information

**DOI:** 10.1001/jamanetworkopen.2026.39482

**Published:** 2026-09-24

In the Original Investigation titled “Association of L-α Glycerylphosphorylcholine With Subsequent Stroke Risk After 10 Years,” published November 24, 2021,^1^ there were errors in the Methods and Article Information sections. In the Methods, the *ICD-10* code given as I61 for ischemic stroke should have been I63, and the *ICD-10* code given as I63 for hemorrhagic stroke should have been I61. In the Author Contributions section of Article Information, the name given as “Author x” should have been “Drs Choi and Park.” This article has been corrected.^1^
