# Supplementary material for: Association of L-α Glycerylphosphorylcholine With Subsequent Stroke Risk After 10 Years
Source: JAMA Netw Open. 2021 Nov 24;4(11):e2136008. doi: 10.1001/jamanetworkopen.2021.36008 (PMC8613599; doi:10.1001/jamanetworkopen.2021.36008)
Supplement: Supplement. — eTable 1. Hazard Ratios for Stroke According to α-GPC Use With Competing Risk Analysis Via Cause-Specific Hazard Model Regression eFigure. Hazard Ratios for Stroke According to α-GPC Prescription Amount Among Those Who Were Prescribed α-GPC With Competing Risk Analysis Via Cause-Specific Hazard Model Regression eTable 2. Hazard Ratios for Stroke According to α-GPC Use After 1:1 Exact Matching eTable 3. Hazard Ratios for Stroke per 1 Interquartile Range Increase in α-GPC Prescription Days Among Those Who Were Prescribed α-GPC [file jamanetwopen-e2136008-s001.pdf]

Supplemental Online Content

Lee G, Choi S, Chang J, et al. Association of L-α glycerylphosphorylcholine with subsequent stroke risk after 10 years. *JAMA Netw Open.* 2021;4(11):e2136008. doi:10.1001/jamanetworkopen.2021.36008

eTable 1. Hazard Ratios for Stroke According to α-GPC Use With Competing Risk Analysis Via Cause-Specific Hazard Model Regression

eFigure. Hazard Ratios for Stroke According to α-GPC Prescription Amount Among Those Who Were Prescribed α-GPC With Competing Risk Analysis Via Cause-Specific Hazard Model Regression

eTable 2. Hazard Ratios for Stroke According to α-GPC Use After 1:1 Exact Matching

eTable 3. Hazard Ratios for Stroke per 1 Interquartile Range Increase in α-GPC Prescription Days Among Those Who Were Prescribed α-GPC

This supplemental material has been provided by the authors to give readers additional information about their work.

© 2021 Lee G et al. *JAMA Network Open*.

Supplemental Table 1. Hazard ratios for stroke according to alpha-GPC use with competing risk analysis via cause specific hazard model regression.

	Total		Men		Women	
	**Non-user**	**User**	**Non-user**	**User**	**Non-user**	**User**
Total cohort						
Total stroke						
Events	745,589	14,138	362,154	5,426	383,435	8,712
Person-years	107,830,473	870,174	49,775,056	295,203	58,055,417	574,970
aHR (95% CI)	1.00 (reference)	1.46 (1.43-1.48)	1.00 (reference)	1.56 (1.52-1.60)	1.00 (reference)	1.39 (1.36-1.42)
Ischemic stroke						
Events	446,469	8,342	233,444	3,311	213,025	5,031
Person-years	107,830,473	870,174	49,775,056	295,203	58,055,417	574,970
aHR (95% CI)	1.00 (reference)	1.36 (1.33-1.39)	1.00 (reference)	1.44 (1.39-1.49)	1.00 (reference)	1.30 (1.27-1.34)
Hemorrhagic stroke						
Events	69,376	1,089	35,767	413	33,609	676
Person-years	107,830,473	870,174	49,775,056	295,203	58,055,417	574,970
aHR (95% CI)	1.00 (reference)	1.36 (1.28-1.44)	1.00 (reference)	1.38 (1.25-1.52)	1.00 (reference)	1.34 (1.24-1.44)
Matched cohort						
Total stroke						
Events	101,067	14,056	36,623	5,407	64,444	8,649
Person-years	8,939,584	867,451	3,072,198	294,503	5,867,386	572,948
aHR (95% CI)	1.00 (reference)	1.44 (1.42-1.47)	1.00 (reference)	1.56 (1.51-1.60)	1.00 (reference)	1.38 (1.35-1.41)
Ischemic stroke						
Events	63,895	8,295	24,185	3,299	39,710	4,996
Person-years	8,939,584	867,451	3,072,198	294,503	5,867,386	572,948
aHR (95% CI)	1.00 (reference)	1.35 (1.32-1.38)	1.00 (reference)	1.45 (1.40-1.50)	1.00 (reference)	1.29 (1.25-1.33)
Hemorrhagic stroke						
Events	8,139	1,083	3,102	412	5,037	671
Person-years	8,939,584	867,451	3,072,198	294,503	5,867,386	572,948
aHR (95% CI)	1.00 (reference)	1.41 (1.32-1.50)	1.00 (reference)	1.43 (1.29-1.58)	1.00 (reference)	1.39 (1.29-1.51)

© 2021 Lee G et al. *JAMA Network Open*.

Adjusted hazard ratios calculated by cause specific hazard model regression after adjustments for age, sex, household income, and Charlson comorbidity index.
Competing event for total stroke included death.
Competing event for ischemic stroke included death and hemorrhagic stroke.
Competing event for hemorrhagic stroke included death and ischemic stroke.
Acronyms: aHR, adjusted hazard ratio; CI, confidence interval.

© 2021 Lee G et al. *JAMA Network Open*.

Supplemental Figure 1. Hazard ratios for stroke according to alpha-GPC prescription amount among those who were prescribed alpha-GPC with competing risk analysis via cause specific hazard model regression.

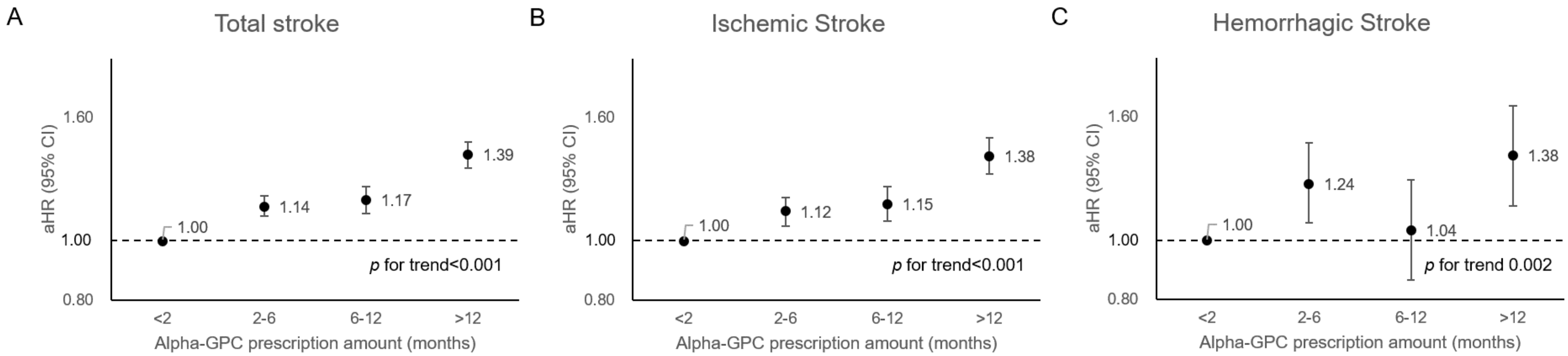

Adjusted hazard ratios calculated by cause specific hazard model regression after adjustments for age, sex, household income, and Charlson comorbidity index. Acronyms: alpha-GPC, L-alpha glycerylphosphorylcholine; aHR, adjusted hazard ratio; CI, confidence interval.

© 2021 Lee G et al. *JAMA Network Open*.

Supplemental Table 2. Hazard ratios for stroke according to alpha-GPC use after 1:1 exact matching.

	Total		Men		Women	
	**Non-user**	**User**	**Non-user**	**User**	**Non-user**	**User**
Number of participants	108,721	108,721	38,779	38,779	69,962	69,9642
Total stroke						
Events	10,198	14,119	3,644	5,418	6,554	8,701
Person-years	895,689	868,874	307,263	294,758	588,426	574,116
aHR (95% CI)	1.00 (reference)	1.43 (1.39-1.47)	1.00 (reference)	1.55 (1.49-1.62)	1.00 (reference)	1.36 (1.32-1.41)
Ischemic stroke						
Events	6,484	8,332	2,435	3,307	4,049	5,025
Person-years	895,689	868,874	307,263	294,758	588,426	574,116
aHR (95% CI)	1.00 (reference)	1.33 (1.29-1.37)	1.00 (reference)	1.42 (1.35-1.50)	1.00 (reference)	1.27 (1.22-1.33)
Hemorrhagic stroke						
Events	834	1,087	316	411	518	676
Person-years	895,689	868,874	307,263	294,758	588,426	574,116
aHR (95% CI)	1.00 (reference)	1.35 (1.23-1.48)	1.00 (reference)	1.36 (1.17-1.57)	1.00 (reference)	1.34 (1.20-1.51)

Alpha-GPC non-users were matched with users via 1:1 exact matching for age, sex, household income, and Charlson comorbidity index.
Adjusted hazard ratios calculated by Cox proportional hazards regression after adjustments for age, sex, household income, and Charlson comorbidity index.
Acronyms: aHR, adjusted hazard ratio; CI, confidence interval.

© 2021 Lee G et al. *JAMA Network Open*.

Supplemental Table 3. Hazard ratios for stroke per 1 interquartile range increase in alpha-GPC prescription days among those who were prescribed alpha-GPC.

	**Total stroke**	**Ischemic stroke**	**Hemorrhagic stroke**
Number of subjects	108,721	108,721	108,721
Events	14,119	8,332	1,087
Person-years	868,874	868,874	868,874
aHR (95% CI)	1.09 (1.07-1.10)	1.09 (1.07-1.10)	1.06 (1.02-1.11)
p value	<0.001	<0.001	0.004

Adjusted hazard ratios calculated by Cox proportional hazards regression after adjustments for age, sex, household income, and Charlson comorbidity index.
Acronyms: aHR, adjusted hazard ratio; CI, confidence interval.

© 2021 Lee G et al. *JAMA Network Open*.
